# WDR5 serves in co-activation and influences genome targeting of KLF3

**DOI:** 10.1093/nar/gkaf977

**Published:** 2025-09-30

**Authors:** Lu Yang, Manan Shah, Tanit Chavalit, Annalise M Spek, Wooi F Lim, Ling Zhong, Vala Nejad Safari, Mahdi Haddad, Thu H Vu, Joel P Mackay, Mark J Raftery, Marc R Wilkins, Jacqueline M Mathews, Kate G R Quinlan, Merlin Crossley

**Affiliations:** School of Biotechnology and Biomolecular Sciences, UNSW Sydney, Sydney, NSW 2052, Australia; School of Biotechnology and Biomolecular Sciences, UNSW Sydney, Sydney, NSW 2052, Australia; School of Biotechnology and Biomolecular Sciences, UNSW Sydney, Sydney, NSW 2052, Australia; School of Biotechnology and Biomolecular Sciences, UNSW Sydney, Sydney, NSW 2052, Australia; School of Biotechnology and Biomolecular Sciences, UNSW Sydney, Sydney, NSW 2052, Australia; Bioanalytical Mass Spectrometry Facility, UNSW Sydney, Sydney, NSW 2052, Australia; School of Biotechnology and Biomolecular Sciences, UNSW Sydney, Sydney, NSW 2052, Australia; School of Biotechnology and Biomolecular Sciences, UNSW Sydney, Sydney, NSW 2052, Australia; School of Life and Environmental Sciences, University of Sydney, NSW 2006, Australia; School of Life and Environmental Sciences, University of Sydney, NSW 2006, Australia; Bioanalytical Mass Spectrometry Facility, UNSW Sydney, Sydney, NSW 2052, Australia; School of Biotechnology and Biomolecular Sciences, UNSW Sydney, Sydney, NSW 2052, Australia; School of Life and Environmental Sciences, University of Sydney, NSW 2006, Australia; School of Biotechnology and Biomolecular Sciences, UNSW Sydney, Sydney, NSW 2052, Australia; School of Biotechnology and Biomolecular Sciences, UNSW Sydney, Sydney, NSW 2052, Australia

## Abstract

Krüppel-like factor 3 (KLF3) is a member of the archetypal SP/KLF family of transcription factors that bind GC-rich elements and CACCC boxes in promoters and enhancers via three classical zinc fingers (ZFs) at or near their C-termini. KLF3 can both repress and activate transcription. It represses by recruiting CtBP co-repressors via its N-terminal domain but the mechanism by which it activates was unknown. Here, we show that KLF3 associates with WDR5 and this interaction is associated with gene activation. We also demonstrate that this interaction is required for proper genomic targeting of KLF3. This helps explain previous results indicating that both the C-terminal ZF DNA-binding domain and N-terminal functional domain are required for KLF3 to identify its target genes. This result adds to only a handful of examples that transcriptional co-regulators, in addition to facilitating activation and repression, can also influence target gene selection.

## Graphical Abstract

**Graphical Abstract:**
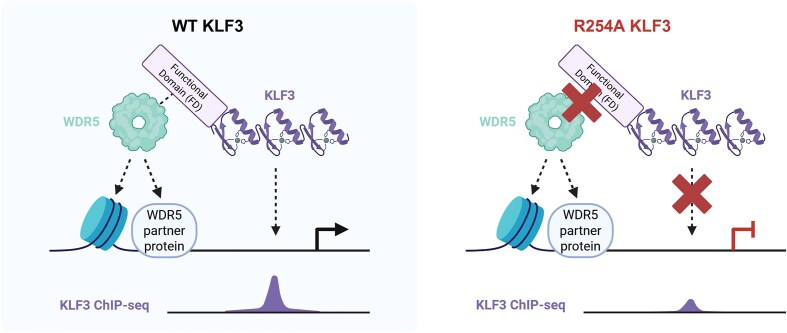

## Introduction

Transcription factors are often considered to be composed of two components: a sequence-specific DNA-binding domain (DBD) and a functional (activation/repression) domain (FD). The localization of the transcription factor to its target genes is usually attributed to the DBD, since it can bind directly to specific sequences in DNA. The FD is considered to control activation or repression of the target gene [1].

We have focussed on one member of the archetypal SP/KLF family, Krüppel-like factor 3 (KLF3) [2, 3]. SP/KLF family members contain a conserved classical Cys2–His2 zinc finger DNA-binding domain (made up of three tandem zinc fingers), at or near their C-terminus, that binds to CACCC- and GC-rich motifs [4–6]. KLF3 is expressed in most organs and multiple cell types. It has been extensively studied in erythroid cells where it is primarily, and perhaps exclusively, a repressor but in other cells it can act as either an activator or repressor [7–9]. The mechanism by which KLF3 activates transcription is unknown but is addressed in this study.

There is accumulating evidence that DBDs alone are not necessarily sufficient for the localization of transcription factors to their target genes. From a theoretical perspective, the DNA-recognition elements for transcription factors are typically very short (often 5–8 nucleotides) and transcription factors only localize to a subset of potential binding sites in the genome. In the case of KLF3, we have found that the DBD of KLF3 is not sufficient for proper localization of KLF3 to all its target genes [9, 10]. Loss of function experiments demonstrated that the FD (KLF3 1–262) also has a key role in localizing KLF3 to certain target genes [9]. Furthermore, gain-of-function experiments established that the FD can directly target an unrelated DBD to loci normally bound by KLF3 [10]. Nevertheless, the mechanism by which the FD mediates localization remained undetermined.

To investigate how the FD serves to localize the protein to specific target genes, and with a view to revealing co-activators of transcription, we sought to identify partner proteins of the FD (non-DNA-binding domain) of KLF3. We constructed a cell line stably expressing an epitope-tagged KLF3 FD and performed co-immunoprecipitation coupled mass spectrometry (CoIP/MS) to identify partner proteins. Encouragingly, we isolated a previously known partner, CtBP [8], and also detected a previously unrecognized KLF3 partner—WD repeat domain 5 (WDR5).

Detailed mapping of the region of KLF3 bound by WDR5 enabled us to create a KLF3 point mutant that was impaired in its association with WDR5. ChIP (chromatin immunoprecipitation)-seq experiments demonstrated that disrupting the interaction of KLF3 and WDR5 significantly affected the localization of KLF3 to a subset of its targets. RNA-seq revealed that the majority of the mis-regulated genes that were bound by KLF3 and WDR5 relied on these proteins for activation, thus establishing WDR5 as the first co-activator found to associate with KLF3. Accordingly, the association with WDR5 helps explain both the activation function and genomic localization of KLF3.

Taken together, our results identify WDR5 as a co-activator of KLF3 that acts not only in activating specific KLF3 target genes but also more fundamentally in localizing KLF3 to a subset of its targets. This has implications for our understanding of how transcription factors identify their target genes in the context of the chromatin landscape, and suggests that different co-regulators, as well as serving a role in activation or repression, also direct transcription factors to different target genes.

## Materials and methods

### Cell culture

Human embryonic kidney (HEK293) cells, mouse embryonic fibroblast (MEF) cells, and COS-1 monkey kidney (COS) cells were cultured in Dulbecco’s modified Eagle’s medium (DMEM; Life Technologies, CA, USA) supplemented with 1% (v/v) penicillin–streptomycin–glutamine (PSG) solution (Life Technologies, CA, USA) and 10% (v/v) fetal bovine serum (FBS; Life Technologies, CA, USA). Cells were grown at 37°C in a 5% CO_2_ water-jacketed incubator.

### Cell transient transfection

COS cells were transiently transfected with pMT3-HA-KLF3 plasmid, pMT3-FLAG-WDR5 plasmid, and various deletion constructs using the FuGENE^®^6 Transfection system. 2 × 10^6^ COS cells were seeded in 10-cm diameter cell culture dishes one day prior to the transfection in DMEM medium supplemented with 1% PSG and 10% FBS. On the day of transfection, 6 μl of FuGENE^®^6 Transfection Reagent (Promega, Cat# E2691) was diluted in 94 μl of DMEM medium without FBS and incubated at room temperature for 10 min. After the incubation, the pre-warmed FuGENE^®^6 Transfection Reagent mixture was mixed with 2 μg of total purified plasmid DNA [1 μg of pMT3-FLAG-WDR5 and 1 μg of pMT3-HA-KLF3 (wild type or various deletion constructs)]. The FuGENE^®^6 Transfection Reagent–DNA mixture was incubated at room temperature for 30 min before being gently added to the COS cells. The transfected cells were incubated at 37°C with 5% CO_2_ for 48 h before being harvested.

### Generation of stable cell lines (retroviral transduction)

The *Klf3^−/−^* murine embryonic fibroblast (MEF) cell line was generated from *Klf3^−/−^* mice as previously described [11] and have been previously reported [9]. Wild-type mouse KLF3-V5 and R254A mutant mouse KLF3-V5 were cloned into a retroviral expression system pMSCVpuro (Clontech Laboratories, CA, USA) to generate stable MEF cell lines expressing KLF3-V5 proteins. The Ecopack™ 2-293 cell line was used as the packaging cell line. pMSCVpuro plasmids were transfected into Ecopack™ 2-293 cells using the FuGENE^®^6 system. Puromycin selection (2.5 μg/ml) was initiated in the MEF cells at 48 h following the viral transduction. Cells were serially diluted into a 150-cm dish and single clonal populations were picked and validated through western blot using an antibody to the V5 tag.

### Co-immunoprecipitation for mass spectrometry

HEK293 stable cell lines [10] were used for this experiment. The cell line stably expressed a V5-epitope-tagged human KLF3 FD fragment or empty vector. Cells were washed with cold phosphate buffered saline (PBS) and pelleted by centrifuging at 300 × *g* for 4 min. Cell pellets were resuspended with cold buffer A (10 mM Hepes, pH 7.8, 1.5 mM MgCl_2_, 10 mM KCl, 1 mM Phenylmethylsulfonyl fluoride (PMSF), 5 mM Dithiothreitol (DTT), 10 μg/ml leupeptin, 10 μg/ml aprotinin) and incubated on ice for 5 min. The lysates were gently vortexed for 20 s to release the nuclei and centrifuged at 228 × *g* for 5 min at 4°C to pellet the nuclei. The supernatant was retained as the cytoplasmic fraction. The nuclear pellets were resuspended in 3 ml of S1 (0.25 M sucrose, 10 mM MgCl_2_) and layered over a 3 ml cushion of buffer S3 (0.88 M sucrose, 0.5 mM MgCl_2_). The mixture was centrifuged at 2800 × *g* for 10 min at 4°C and the supernatant was discarded. The nuclear pellets were resuspended in 5 ml cold RIPA buffer (50 mM Tris, pH 7.5, 150 mM NaCl, 1% NP-40). The mixture was sonicated for 10 s on/off, five times at medium amplitude (Diagenode) at 4°C. The lysate was centrifuged at 2800 × *g* for 10 min at 4°C. The supernatant was either used immediately for co-immunoprecipitation (CoIP) or was stored at −80°C. Hundred microlitres of the supernatant was kept as input. Approximately 1 ml of supernatant was precleared with pre-washed protein G dynabeads (Life Technologies, CA, USA, Cat# 10004D) by gently rocking at 4°C for 30 min. The supernatant–bead mixture was placed on the magnetic stand for 30 s and the pre-cleared supernatant was then collected into a separate tube. The pre-cleared supernatant was incubated with 24 μg of anti-mouse-V5 antibody (Life Technologies, CA, USA) by gently rocking at 4°C for 30 min. Hundred microlitres of the Dynabeads protein G (Life Technologies, CA, USA) were equilibrated twice with 1 ml cold RIPA buffer (50 mM Tris, pH 7.5, 150 mM NaCl, 1% NP-40). The pre-washed beads were added to the pre-cleared supernatant–antibody mixture and incubated at 4°C for 30 min. The supernatant–antibody–bead mixture was washed with 1 ml of low salt RIPA buffer (50 mM Tris, pH 7.5, 75 mM NaCl, 1% NP-40) four times and washed with 1 ml of MiliQ water twice. The washed supernatant–antibody–bead mixture was proceeded to the elution step. The bound proteins were eluted from the beads with 150 μl of 0.1 M glycine (pH 2.6) by incubating at 65°C for 15 min. The eluate was collected, and the pH was neutralized to 7–9 by adding 15–20 μl of 1 M Tris (pH 11). The eluate was incubated with 5 mM DTT at 37°C for 30 min. Iodoacetamide (Sigma–Aldrich) was added to a final concentration of 10 mM. The eluate was covered with aluminium foil and incubated at 37°C for 30 min. After incubating with iodoacetamide, the eluate was incubated with 5–10 ng/μl of trypsin (Sigma–Aldrich) at 37°C for 14 h such that it was ready for mass spectrometry.

### Mass spectrometry analysis

The trypsinized eluate was used to perform mass spectrometry at the Bioanalytical Mass Spectrometry Facility, UNSW. Digested peptides were separated by nano-LC using an UltiMate 3000 UPLC and autosampler system (Thermo Fisher Scientific, San Jose, CA, USA). Samples (2.5 μl) were concentrated and desalted onto a micro C18 precolumn (300 μm × 5 mm; Thermo) with H_2_O:CH3CN (98:2, 0.1% trifluoroacetic acid (TFA)) at 15 μl/min. After a 4-min wash, the pre-column was switched (Valco 10 port valve, Valco, Houston, TX) into line with a fritless nano column (75 μm × ∼15 cm) containing C18AQ media (1.9 μm, 120 Å Dr Maisch, Ammerbuch-Entringen, Germany). Peptides were eluted using a linear gradient of H_2_O:CH3CN (98:2, 0.1% formic acid) to H_2_O:CH3CN (64:36, 0.1% formic acid) at 200 nl/min over 30 min. High voltage of 2000 V was applied to low volume union (Valco) and the column tip positioned at ∼0.5 cm from the heated capillary (T = 275°C) of an Orbitrap Velos Pro (Thermo Electron, Bremen, Germany) mass spectrometer. Positive ions were generated by electrospray and the Orbitrap operated in data-dependent acquisition mode.

A survey scan of *m*/*z* 350–1750 was acquired in the Orbitrap (resolution = 30 000 at *m*/*z* 400, with an accumulation target value of 1 000 000 ions) with lockmass enabled. Up to the 10 most abundant ions (>5000 counts) with charge states >+2 were sequentially isolated and fragmented within the linear ion trap using collisional induced dissociation with an activation *q* = 0.25 and activation time of 10 ms at a target value of 30 000 ions. *m*/*z* ratios selected for MS/ MS were dynamically excluded for 30 s.

Peak lists were generated using Mascot Daemon/extract_msn (Matrix Science, London, UK, Thermo) using the default parameters, and submitted to the database search program Mascot (version 2.3, Matrix Science). Search parameters were as follows: precursor tolerance 4 ppm and product ion tolerances ±0.4 Da; met(O) carboxyamidomethyl-Cys specified as variable modification; enzyme specificity was trypsin; one missed cleavage was possible; and the non-redundant protein database from SwissProt (Jan 2016) searched.

Mascot search results were analysed with Scaffold (Proteome software). Protein identifications were selected with this criteria: 99% protein threshold, three minimum peptides, and 95% peptide threshold.

### Co-immunoprecipitation

COS cells transfected with pMT3-HA-KLF3 plasmid, pMT3-FLAG-WDR5 plasmid, and various deletion constructs were used in this experiment. Transfected cells were harvested after 48 h. Monolayer cells were washed with 1 ml cold PBS and centrifuged at 300 × *g* for 5 min. The cell pellet was resuspended and incubated in 250 μl of NP-40 lysis buffer [50 mM Tris, pH 8.0, 150 mM NaCl, 1% (v/v) NP-40, 1 mM PMSF, 10 μg/ml leupeptin, and 10 μg/ml aprotinin] on ice for 30 min. The cell suspension was centrifuged at 13 000 × *g* for 10 min at 4°C. The supernatant was collected as a whole cell extract. Ten percent of the whole cell extracts was used as input for the CoIP. The remaining 90% of the whole cell extracts was incubated with 40 μl of pre-washed agarose beads (Sigma–Aldrich, Cat# A0919) by gently rocking at 4°C for 30 min. The lysate–bead mixtures were centrifuged at 7000 × *g* for 30 s to collect the pre-cleared lysate. The pre-cleared lysate was incubated with 40 μl of FLAG-M2 agarose beads (Sigma–Aldrich, Cat# 2220) by gently rocking at 4°C for 1 h. The FLAG-M2 agarose beads were washed with 400 μl NP-40 lysis buffer four times and spun down at 7000 × *g* for 30 s. After the last spin, 20 μl of protein loading dye (Thermo Fisher Scientific, Cat# NP0007) was added to the beads and boiled at 100°C for 3 min such that they were ready for western blot analysis.

### Western blot

Nuclear extracts were made as previously described [12]. Sixty micrograms of cell nuclear extract was loaded onto a NuPAGE™ Novex™ 10% Bis–Tris Protein Gel (Thermo Fisher Scientific, Cat# NP0301BOX) and samples were run at 200 V for 60 min. Proteins were transferred on to a nitrocellulose membrane at 30 V for 60 min. Blots were blocked using 4% skim milk in Tris-buffered saline with 0.1% Tween^®^20 detergent (TBST) for 30 min. After blocking, blots were probed with primary antibodies. We used anti-FLAG antibody (Sigma–Aldrich, Cat# F3165) and anti-HA antibody (Sigma–Aldrich, Cat# H3663) to check the protein levels of KLF3 and WDR5 after CoIP. We used anti-V5 antibody *(*Life Technologies, Cat# R960CUS) and anti-ACTIN antibody (Sigma–Aldrich, Cat# A1987) to test the KLF3 expression level in MEF stable cells.

### Chromatin immunoprecipitation

ChIP experiments were performed as previously described [11]. Approximately, 7 × 10^7^–1 × 10^8^ cells were used for each ChIP. Cells were cross-linked with 1% formaldehyde for 10 min and the reaction was quenched by 2.5 mM glycine. Fixed cells were washed with cold PBS and centrifuged at 2000 × *g* for 5 min. Fixed cells were sonicated at high voltage for 20 min (30 s on, 30 s off) using a Bioruptor (Diagenode) to obtain 200–300 bp DNA fragments. The fragmented chromatin was pulled down at 4°C overnight using an antibody against V5 (10 μg; Life Technologies, #R960CUS) or WDR5 (15 μg;Australian Biosearch, #13105S). Chromatin cross-linking was then reversed at 65°C overnight, followed by DNA purification. Real-time quantitative polymerase chain reaction (qPCR) was performed on ChIP DNA on an Applied Biosystems ViiA7 Real-time PCR System (Applied Biosystems) to confirm the success of the ChIP experiments before sending to next-generation sequencing.

### DNA library preparation and next-generation sequencing

DNA library preparation was performed using the TruSeq DNA Sample Preparation Kit (Illumina, Cat# FC-121-2001) according to the manufacturer’s instructions. Adapter sequences were diluted 1/40 before use and following adapter ligation, the library size extracted from the gel was 100–280 bp (excluding adapters) in line with the size of sonicated fragments. Libraries from eight samples were multiplexed into four lanes such that there were two samples per lane. Samples were sequenced using 75 bp read chemistry on the NextSeq 500 (Illumina). Sequencing was performed by the Ramaciotti Centre for Genomics, an internal core sequencing facility at UNSW Sydney.

### RNA extraction

Total RNA was extracted from wildtype KLF3 MEF cells and R254A mutant KLF3 MEF cells with TRI Reagent according to the manufacturer’s protocol (Qiagen, Cat# 74004). Two-hundred microlitres of chloroform was added to 1 ml TRI Reagent and vortexed for 10 s. The mixture was spun at 12 000 × *g* for 5 min. The aqueous phase was transferred into a new Eppendorf tube. The aqueous phase was mixed with an equal volume of ethanol. The RNA–ethanol mixture was added to the column and washed once with wash buffer. RNA retained in the column was treated with DNA Removal Kit (Qiagen, Cat# 79254) to ensure the purity of RNA samples. After DNase treatment, bound RNA was washed with wash buffer and then eluted with RNase A free water. RNA concentration was determined by UV-light absorbance at 260 nm using a NanoDrop machine (Thermo Fisher Scientific). The integrity of RNA was checked by Bioanalyzer (Agilent).

### RNA library preparation and next-generation sequencing

Coding RNA was pulled down by virtue of the poly A tail and was converted into a library of template molecules of known strand origin using the TruSeq Stranded mRNA Sample Preparation Kits *(*Illumina, Cat# RS-122-2101) according to the manufacturer’s instructions. Multiple indexing adapters were ligated to the ends of the double-stranded complementary DNA (cDNA). cDNA fragments that had adapter molecules on each end were amplified with a minimum number of PCR (polymerase chain reaction) cycles to avoid skewing the representation of the library. Samples were sequenced using 75 bp single read chemistry on the NextSeq 500 (Illumina). Sequencing was performed by the Ramaciotti Centre for Genomics.

### ChIP-seq analysis

After quality filtering and adapter trimming using Trimmomatic v0.3.3 using the default parameters (LEADING:3 TRAILING:3 SLIDINGWINDOW:4:15 MINLEN:36) [13], reads were aligned to the mm10/GRCm38 *Mus musculus* genome using Bowtie v2.2.9 [14] set to ‘very sensitive’. Aligned reads were filtered using SAMtools v1.3.1 [15] to remove unmapped reads, non-primary alignments, and alignments with a mapping quality <10 (-F 1804 -q 10).

Peak calling for all datasets was done using the SPP package [16, 17] after removing duplicates with Picard MarkDuplicates v1.109. A set of consistent peaks (Irreproducible Discovery Rate (IDR) = 0.01 for KLF3-V5 or 0.05 for WDR5) were then identified using IDR2 [17, 18]. Peak lists were annotated using HOMER v4.8 [19] with the GENCODE vM20 annotation. Genomic localization analysis was performed using ChIPseeker [20]. Peak overlaps were performed using BEDTools intersect [21]. To perform differential binding analysis, filtered (but not de-duplicated) alignment files along with IDR peak files were used as inputs for DiffBind v3.0.8 [22]. For peak merging and counting reads, the default parameters were used. A false discovery rate (FDR) value of 0.05 was used to identify significantly differentially bound peaks. For volcano plots, the log_2_ fold change and FDR values were obtained from the DiffBind result tables. *De novo* motif discovery was performed using MEME-ChIP v5.0.2 (-meme-nmotifs 5 -meme-maxw 25) [23] on 200 bp regions around the centre of the passed peak sets.

Read coverage tracks were generated using deepTools bamCoverage [24] with the filtered mapped reads, extending reads based on predicted fragment sizes from SPP, and normalizing all samples to have the equivalent of 1 million mapped reads (-bs 1 --normalizeUsing CPM). The resulting bigwig files were then visualized on IGV.

For average summary plots, computeMatrix followed by plotProfile from deepTools [24] was used. For KLF3 summary plots, computeMatrix was passed using default parameters with –maxThreshold set to 1000.

### RNA-seq analysis

Raw FASTQ files were quantified using Salmon v1.6.0 [25] in selective alignment mapping mode (–validateMappings –rangeFactorizationBins 4) using a GENCODE vM20 [26] (based on *Mus musculus* mm10 genome). A decoy-aware index was built by concatenating the GRCm38 primary assembly genome to the end of the GENCODE vM20 transcriptome. Count files were then processed using tximport [27] to obtain gene-level counts. Differential gene expression analysis was performed using DESeq2 [28] using a log_2_ fold change threshold of 1.5 and an adjusted *P*-value of <0.05. Heatmaps were generated using the R package pheatmap.

### ChIP-seq and RNA-seq combined analysis

For the combined ChIP-seq and RNA-seq analysis, the KLF3 and WDR5 DiffBind peaksets were used. Using BEDTools intersect, these peaksets were then overlapped with each other and a BED file of all promoters (−1000 to +1000 bp from the transcription start site (TSS) of any known transcript) generated using the gencode vM20 comprehensive gtf annotation file. This set of peaks with corresponding promoter and gene information was then combined with the RNA-seq data using R. This dataset that contained peak and differential binding information, KLF3 and WDR5 overlap information, and RNA-seq data was then filtered according to the different scenarios tested in Fig. 6.

## Results

### WDR5 associates with the KLF3 functional domain

**Figure 1:**
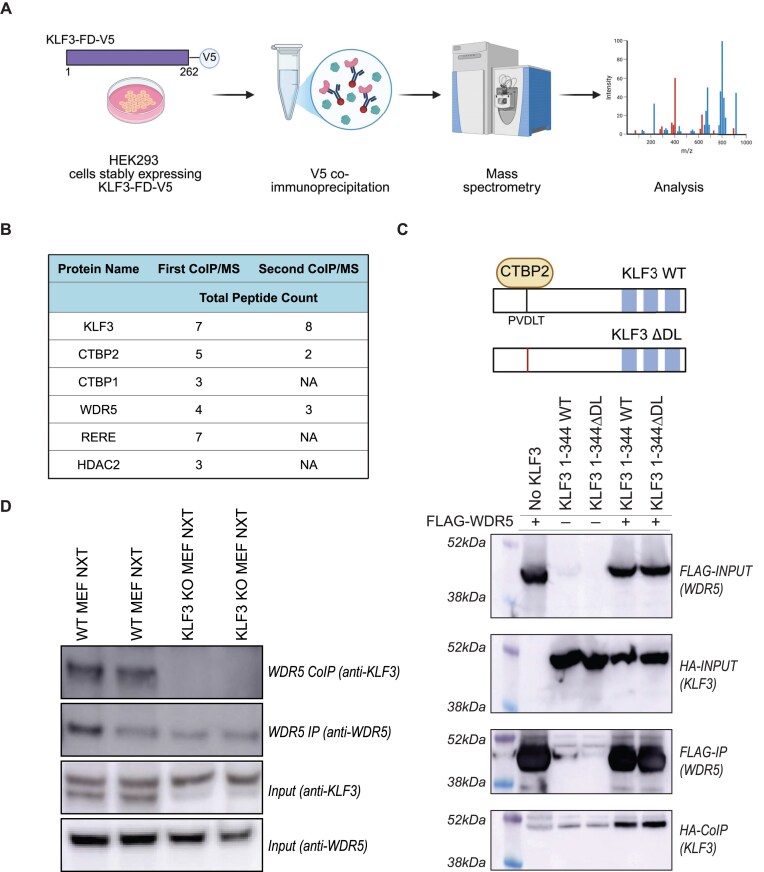
Identification of WDR5 as a KLF3 partner protein. (**A**) CoIP coupled with mass spectrometry (CoIP/MS) in HEK293 cells. (**B**) Selected candidate proteins found in CoIP/MS from HEK293 cells stably expressing KLF3-FD-V5. The total peptide count represents the number of unique peptides mapped to the protein in each the CoIP/MS experiments. Peptides found in the negative control empty vector cells were disregarded. (**C**) CoIP experiments were performed in COS cells with recombinant wild-type KLF3 (1–344) or mutant KLF3 (1–344 ΔDL). Sigma M2 FLAG agarose beads were used to pull down the FLAG-WDR5. The immunoprecipitated FLAG-WDR5 and HA-KLF3 were detected with FLAG and HA antibodies, respectively. (**D**) CoIP experiments were performed in WT or KLF3 KO MEF cells with endogenous WT KLF3 using anti-KLF3 or anti-WDR5 antibodies.

To identify partners of the KLF3 FD, we performed CoIP/MS in HEK293 cells stably expressing a V5-epitope-tagged KLF3 FD fragment, KLF3-FD-V5 (1–262) [10]. Nuclear proteins were extracted, checked for V5 antibody immunoprecipitation, and used for CoIP/MS in duplicate experiments (Fig. 1A).

To identify proteins from immunoprecipitations, peptide fragmentation spectra were matched against the human proteome using the Mascot search engine. Background proteins that were detected in the negative control HEK293 line, which did not express KLF3-FD-V5, were discarded. The experiments successfully recovered the well-characterized KLF3 partners, CtBP1 and 2 (Fig. 1B), as well as the co-regulator WDR5. Components of the recently described WHHERE complex, namely RERE and HDAC2 were also identified in one replicate of the CoIP/MS experiments. (A full list of candidate proteins found is available in Supplementary Table S1) [29].

To verify the interaction between KLF3 and WDR5, a number of additional experiments were carried out. Full-length wild-type HA-epitope-tagged KLF3 (KLF3 1–344 WT) or full-length HA-KLF3 containing a mutation at the CtBP interaction site (KLF3 1–344 ΔDL) was co-expressed with FLAG-epitope-tagged WDR5 (FLAG-WDR5) and CoIP assays performed. Both KLF3 1–344 WT and KLF3 1–344 ΔDL were co-immunoprecipitated by FLAG-WDR5 (Fig. 1C). To ensure that the KLF3–WDR5 interaction was not an artefact caused by overexpression, we performed CoIP in WT and KLF3 KO MEF cells. WDR5 was co-immunoprecipitated only in the presence of KLF3 (in WT MEF cells) (Fig. 1D and Supplementary Fig. S1). These results suggest that WDR5 is not recruited to KLF3 via CtBP and that the KLF3–WDR5 association occurs endogenously.

### WDR5 associates via a short motif in the KLF3 functional domain

**Figure 2:**
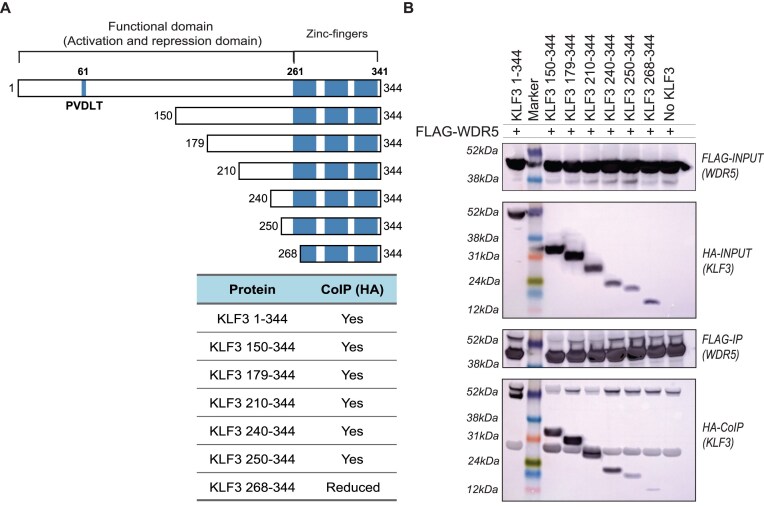
KLF3 N-terminal deletion mapping determines an association interface with WDR5 at KLF3 residues 250–268. The WDR5 association motif in KLF3 was mapped using successive 30 amino acid KLF3 N-terminal deletions from residues 1 to 268 by CoIP. (**A**) Diagram of the truncated KLF3 constructs used for mapping experiments. The table summarizes whether KLF3 still associated with WDR5. (**B**) CoIP experiments were performed in COS cells with overexpressed FLAG-WDR5 and different truncations of HA-KLF3. Sigma M2 FLAG agarose beads were used to pull down FLAG-WDR5 and the ability to co-immunoprecipitate various HA-KLF3 truncations assessed in western blots with FLAG and HA antibodies.

To identify the WDR5 association interface on KLF3, we generated a series of KLF3 deletion constructs and tested their interaction with WDR5, using CoIP assays (Fig. 2A). KLF3 truncated from the N-terminus up to amino acid 250 was still able to bind strongly to WDR5, thereby narrowing the WDR5 interaction interface to KLF3 amino acids 250–344 (Fig. 2). A further N-terminal truncation removing the first 267 amino acids of KLF3 (268–344) disrupted the association of WDR5 with KLF3, suggesting the WDR5 association motif lies within KLF3 amino acids 250–268 (Fig. 2B).

**Figure 3:**
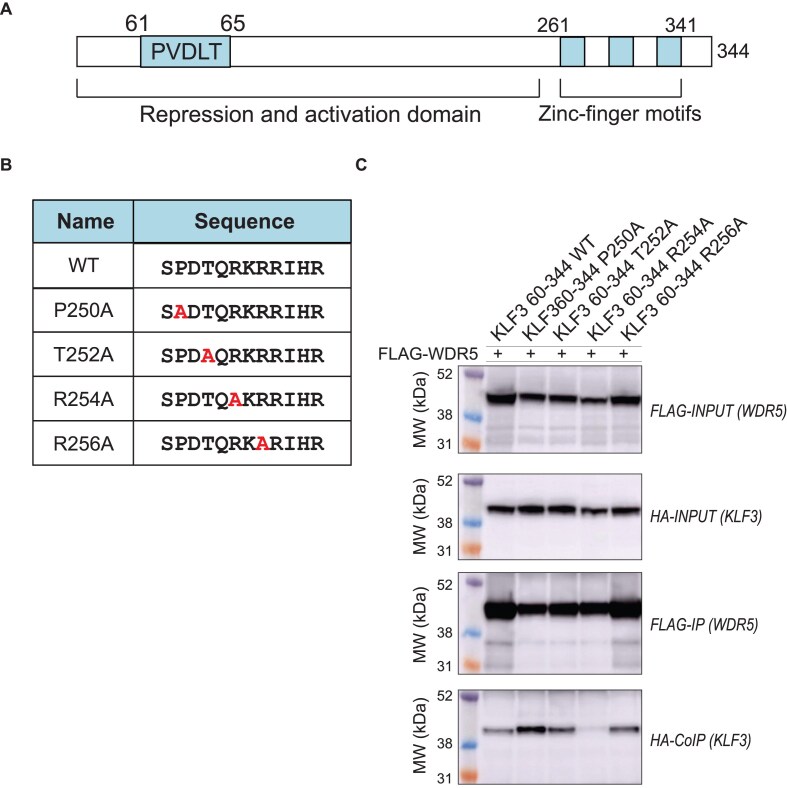
An R254A mutation in KLF3 disrupts association with WDR5. (**A**) Diagram of KLF3 domains. (**B**) Table outlining point mutations at KLF3 residues 250, 252, 254, and 256 that were introduced into KLF3 60–344. (**C**) CoIP was performed in COS cells over-expressing FLAG-WDR5 and HA-KLF3 wild type (60–344), HA-KLF3 P254A, HA-KLF3 T252A, HA-KLF3 R254A, or HA-KLF3 R256A using Sigma M2 FLAG beads. Western blots were probed with FLAG and HA antibodies to detect FLAG-WDR5 and HA-KLF3.

To further define the WDR5 association motif in KLF3 amino acids (Fig. 3A), point mutations were generated across the 250–262 region (Fig. 3B) in the context of a KLF3 fragment encompassing KLF3 (60–344), and the ability of the mutant proteins to bind WDR5 was assessed by CoIP (Fig. 3C). While a number of mutations reduced the interaction, mutation of arginine 254 to alanine caused a marked reduction in WDR5 association. The conservation of this region in KLF3 proteins between species was examined. Alignment of KLF3 in four diverse species (human, mouse, chicken, and frog) shows that the WDR5 association motif lies within a highly conserved region of KLF3 (Supplementary Fig. S2). Together, these data establish that WDR5 is a partner of KLF3 that directly interacts with the KLF3 FD, and that an KLF3 R254A substitution is sufficient to disrupt the association with WDR5.

The amino acid sequence of KLF3 between 251 and 260 was aligned with two previously characterized WDR5 binding motifs: WDR5-binding motif (WBM) [30–32] and WDR5-interacting motif (Win) [33–35]. There were no apparent similarity between KLF3 251–260 residues and these previously identified WDR5 binding motifs (Supplementary Fig. S3), suggesting that KLF3 may interact with WDR5 in a distinct way to that of other known WDR5 partner proteins.

### Disrupting the KLF3/WDR5 association via the R254A mutation causes significant changes in the transcriptome and in genome-wide KLF3 and WDR5 binding

**Figure 4:**
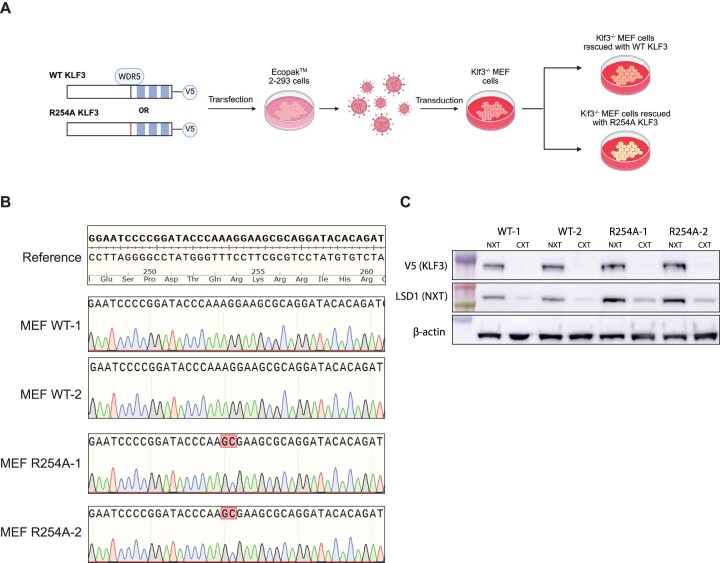
Generation of MEF cells stably overexpressing WT and R254A KLF3. (**A**) Strategy for stable overexpression of V5-tagged wild-type KLF3 and KLF3 R254A mutant in MEF KLF3 knock-out cells. (**B**) The correct introduction of the R254A mutation was confirmed by Sanger sequencing. (**C**) Equivalent expression levels of wild-type KLF3 and R254A KLF3 in two clones stably expressing each construct from the nuclear (NXT) or cytoplasmic extract (CXT) were validated by western blot using an antibody to the V5 epitope tag.

**Figure 5:**
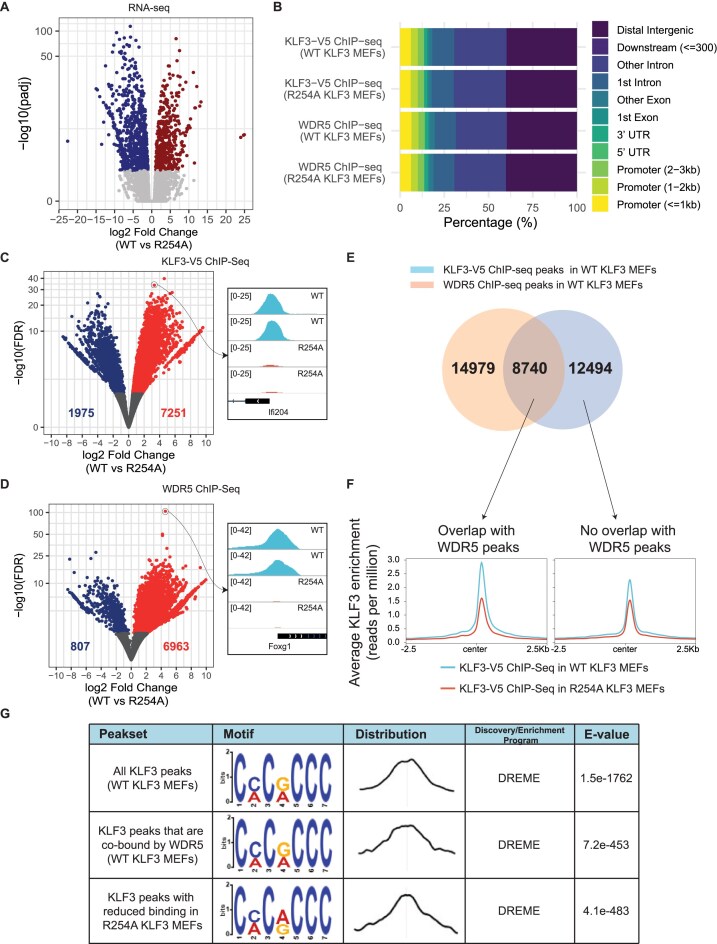
The R254A mutation causes significant transcriptome and genome-wide binding changes to both KLF3 and WDR5. (**A**) A volcano plot showing the fold change (log_2_) and statistical significance [−log(*P*_adj_)] when comparing RNA-seq in WT KLF3 MEFs and R254A KLF3 MEFs. (**B**) The genomic distribution of ChIP-seq peaks for KLF3-V5 and WDR5 in WT KLF3 MEFs and R254A KLF3 MEFs. (**C**) A volcano plot showing the fold change (log_2_ fold change) and statistical significance [−log_10_(FDR)] when comparing KLF3 ChIP-seq peaks in WT KLF3 MEFs versus R254A KLF3 MEFs. (**D**) A volcano plot showing the fold change (log_2_ fold change) and statistical significance [−log_10_(FDR)] when comparing WDR5 ChIP-seq peaks in WT KLF3 MEFs versus R254A KLF3 MEFs. (**E**) Overlap between KLF3-V5 ChIP-seq and WDR5 ChIP-seq peaks in WT KLF3 MEF cells. (**F**) The average KLF3 binding profile (normalized read count) of the KLF3 ChIP-seqs in WT KLF3 MEFs and R254A KLF3 MEFs at KLF3 peaks alone or at peaks co-occupied by WDR5 in WT KLF3 MEFs. Read counts were normalized to the equivalent of a million reads i.e. reads per million. (**G**) *De novo* motif analysis using MEME-ChIP of all KLF3 peaks in WT KLF3 MEFs, KLF3 peaks co-localized by WDR5 in WT KLF3 MEFs, and KLF3 peaks with reduced binding in R254A KLF3 MEFs. The motif distribution around the centre of the peak, the statistical significance of the motif discovered (*E*-value), and the discovery/enrichment program that identified the motif are also shown.

To investigate the functional role of the KLF3/WDR5 interaction, we generated KLF3 knock-out MEF cells stably rescued with either WT KLF3-V5 (WT KLF3 MEFs) or R254A KLF3-V5 (R254A KLF3 MEFs) (Fig. 4A). Successful generation of clones was first confirmed by Sanger sequencing (Fig. 4B). Western blots were also performed to confirm similar expression and nuclear localization (Fig. 4C). We then performed RNA-seq in both lines. The R254A mutation caused significant transcriptome changes when compared to WT KLF3: 1098 genes were differentially regulated (*P*_adj_ < .05) (Fig. 5A).

To investigate which genes were directly bound and thus might be directly regulated by the KLF3/WDR5 complex, we performed KLF3-V5 and WDR5 chromatin immunoprecipitation followed by next-generation sequencing (ChIP-seq) on two clones stably expressing WT KLF3-V5 and two clones stably expressing R254A KLF3-V5 to similar levels (Fig. 4C). We then carried out several ChIP-seq analyses to define the set of genes bound by KLF3 and by WDR5, so that we could match the binding data with the RNA-seq data to assess the effect of abrogating the KLF3/WDR5 interaction via the R254A mutation in KLF3.

In the MEF cells rescued with WT KLF3, ∼21 000 KLF3 ChIP-seq peaks and ∼24 000 WDR5 peaks were identified. We also analysed the genomic localization of both proteins in cells rescued with the KLF3 R254A mutant that cannot bind WDR5 (Fig. 5B). We observed 22 000 peaks corresponding to KLF3 R254 binding, similar to the number bound by WT KLF3, but interestingly, only around 8000 WDR5 peaks were detected. This result suggests that KLF3 is required for the localization of WDR5 to a significant number of potential target genes.

To further assess the effect of the R254A mutation on both KLF3 and WDR5 genome-wide binding, we performed differential binding analysis using DiffBind [22]. The R254A mutation caused significant binding changes with 34% (9226/27 049) of all merged KLF3 and 39% (7770/20 125) of all merged WDR5 peaks being affected. Most differentially bound KLF3 sites showed greater enrichment in WT KLF3 MEFs (7251/9226) (Fig. 5C). This was also true for WDR5 binding with an even higher proportion of differentially bound WDR5 sites displaying greater enrichment in WT KLF3 MEFs (6963/7770), consistent with the reduced number of WDR5 peaks identified in R254A KLF3 MEFs (Fig. 5D). These results suggest that the R254A mutation of KLF3 has a significant influence on both KLF3 and WDR5 genomic localization, with the mutation reducing binding in the vast majority of cases.

We next asked whether KLF3 and WDR5 were co-localized in the genome. A significant proportion, namely about a third of the peaks, contained both KLF3 and WDR5, consistent with the view that KLF3 and WDR5 associate and co-localize at particular loci (Fig. 5E). At these overlapping regions and at regions only bound by KLF3, we examined the average KLF3 binding profile (normalized read count) in both WT KLF3 MEFs and R254A KLF3 MEFs. There was a significant reduction in binding of KLF3 where the R254A mutation was present, with a greater reduction in binding seen in sites co-occupied by WDR5 (Fig. 5F). These results suggest that binding to sites that are co-occupied by KLF3 and WDR5 depends at least in part on the interaction between KLF3 and WDR5, whereas peaks at which only KLF3 is detected are less dependent on or are independent of the KLF3/WDR5 interaction, as expected.

We also performed *de novo* motif analysis using MEME-ChIP [24] on various sets of peaks: KLF3 peaks bound in WT KLF3 MEFs, those especially co-localized with WDR5, and KLF3 peaks that are bound more strongly in WT KLF3 MEFs. A canonical KLF3 motif, namely CACCC, was centrally enriched in all these sets (Fig. 5G). This suggests, perhaps not unexpectedly, that the KLF3 zinc fingers continue to recognize their standard recognition element, irrespective of whether KLF3 is associated with WDR5 or not. Thus, the effect of WDR5 on KLF3 localization to its target genes is not related to any change in DNA-binding specificity of the zinc finger domain of KLF3 but must be related to other phenomena, such as protein–protein interactions made by WDR5.

### The KLF3/WDR5 complex is implicated in gene activation

**Figure 6:**
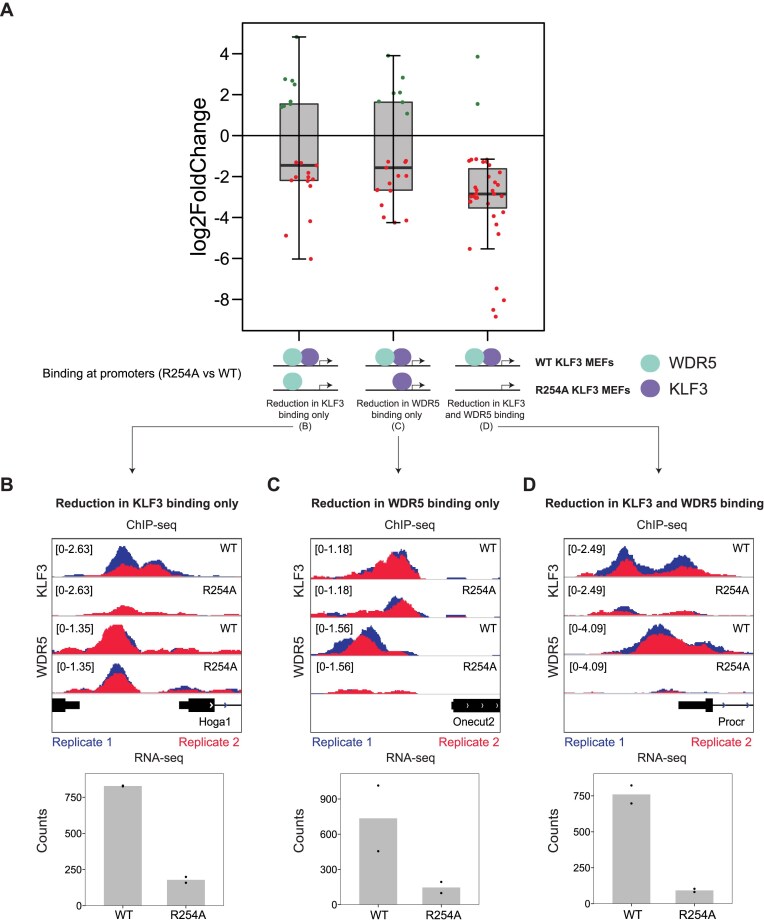
The KLF3/WDR5 complex is associated with gene activation. (**A**) The average fold change (log_2_ fold change) of differentially expressed genes (*P*_adj_ < .05, R254A KLF3 MEFs versus WT KLF3 MEFs) that have both KLF3 and WDR5 bound in their promoter (±1 kb TSS). RNA-seq (normalized counts) and KLF3 and WDR5 ChIP-seq in WT KLF3 MEFs and R254A KLF3 MEFs at genes where the R254A mutation causes downregulation of gene expression and (**B**) a reduction in KLF3 binding only, (**C**) a reduction in WDR5 binding only, and (**D**) a reduction in both KLF3 and WDR5 binding.

To determine the effect on the transcription of genes that are co-occupied by both KLF3 and WDR5 at their promoter (±1 kb TSS), we combined the RNA-seq and ChIP-seq data. We specifically sought to determine the impact of abrogating the contact between KLF3 and WDR5 via the R254A mutation in KLF3. The expression of many genes was unaffected, but we focussed on those genes whose expression was altered and that showed KLF3 and WDR5 peaks in the WT sample. We then divided these genes into three subsets: those where the R254A mutation reduced the KLF3 ChIP-seq peak, those where it reduced the WDR5 peak, and those where both peaks were reduced. We found that when either only KLF3 binding (13/21) or only WDR5 binding (14/21) was reduced, most genes had reduced expression (Fig. 6A). This effect was even more pronounced when both KLF3 and WDR5 binding was reduced by the R254A KLF3 mutation, with 94% of genes being downregulated (33/35) (Fig. 6A). Examples of specific traces for KLF3 and WDR5, together with the drop in expression of representative individual genes, are shown in Fig. 6B–D. A similar trend was observed when considering genes within 10 kb of KLF3/WDR5 binding (Supplementary Fig. S4). Taken together, these results suggest that these genes that are co-occupied by both KLF3 and WDR5 depend on the KLF3/WDR5 complex for activation.

## Discussion

Here, we have identified WDR5 as a functional partner of KLF3. ChIP-seq data indicate that KLF3 and WDR5 co-localize at >8500 sites in MEFs. RNA-seq data demonstrated that at the co-occupied genes whose expression is affected, the majority are reduced in expression when a mutant version of KLF3, R254A, that is unable to contact WDR5, was used. This suggests that the KLF3/WDR5 association facilitates gene activation. This may be consistent with WDR5 serving in co-activation when present as part of the WHHERE complex; however, further validation is needed to determine whether KLF3 associates only with WDR5 or also associates with other members of the WHHERE complex [29]. During ongoing work on this manuscript, an independent study also identified WDR5 as a KLF3 partner in a large screen using a proximity-dependent labelling approach (BioID) [36]. WDR5 was also identified as a partner of KLF12, suggesting that the mechanisms described here for KLF3 may also apply to some other KLF family members.

Most interesting perhaps is the observation that abrogating the interaction between KLF3 and WDR5 via the R254A mutation affected the proper localization of both proteins to the full set of their target genes. The R254A mutation in KLF3 impaired the association of KLF3 with about a third of its genomic target sites. In addition, WDR5 was also lost from about a third of its usual sites. Thus, the protein–protein interaction between WDR5 and KLF3 is required for the proper genomic localization of both proteins to sites that would normally be co-occupied by KLF3 and WDR5. This result fits with previous work that shows that WDR5 is required for the proper localization of c-Myc to its target genes [32]. More generally, the data suggest that WDR5 might play a role in targeting of transcription factors to the genes they regulate, as well as contributing to co-regulatory functions. It also adds to other examples such as that of GATA-1 and its cofactor Friend of GATA-1 (FOG-1). FOG-1 facilitates chromatin occupancy of GATA-1 and a GATA-1 mutant unable to bind FOG-1 localizes to different binding sites [37, 38].

This raises the question of the mechanism by which co-operative targeting operates. Both KLF3 and c-Myc have well-defined sequence-specific DBDs that enable their localization to their recognition sequences in DNA and thus it is clear that they can help localize WDR5 to particular parts of the genome via this simple mechanism. How WDR5, in turn, influences (directs or stabilizes) the genomic targeting of KLF3 (or in other partners, such as c-Myc) is not yet clear. This is in part as we do not yet know whether the KLF3–WDR5 association is direct or indirect (i.e. via both proteins being part of the same multi-protein complex). WDR5 can make numerous protein–protein interactions and could therefore contact various different DNA-bound transcription factors or other co-regulators or regulatory complexes [39]. On the other hand, and perhaps most intriguingly, WDR5 can also act as a histone code ‘reader’ and can bind to H3R2me2 [40] and serotonylated histone H3Q5 [41]. Thus, it is possible that WDR5 may be able to deliver KLF3 to genes decorated with specific chromatin marks. Accordingly, this new perspective might explain why certain transcription factors have been found to bind different sets of target genes in different cell types—because the histone modifications or other transcription factors bound to those genes vary across cell types.

The realization that transcriptional co-regulators, rather than simply being recruited by transcription factors to activate or repress gene expression, might have an active role in contributing to target gene selection offers a perspective on how genes are regulated. Co-regulators can be considered not simply as functional effectors but also as partners in target gene identification that stabilize or direct their partners to target genes. Thus, the availability of co-regulators or the control of their association with transcription factors via signalling pathways and post-translational modifications may not merely determine whether or not a transcription factor can activate or repress its target gene, it may also be decisive in determining whether a transcription factor like KLF3 can even find that gene. Additionally, the association of transcription factors with different co-regulators in different tissues or under different physiological conditions could lead to the binding to different target genes, perhaps helping to explain why even ubiquitous transcription factors can regulate different targets in different cellular environments. Hitherto, it has been generally thought that the binding of transcription factors depended on whether chromatin was ‘open’ or ‘closed’, with pioneer factors required to remodel and open chromatin, but it may be simply that different landscapes of bound proteins are required.

In summary, the historic view of transcription factors was that they contain a DBD that localizes them to their target genes and an FD that recruits co-regulators that activate or repress gene expression. We provide evidence that KLF3 at least, and presumably other transcription factors such as c-Myc and GATA-1, instead work with co-regulators to identify specific sets of target genes partly through DNA–protein interactions but also through protein–protein or protein–histone code binding of specific loci that are decorated by resident protein epitopes or histone marks. The different landscapes in different tissues may explain why even ubiquitous transcription factors bind different genes in different cell types.

## Supplementary Material

gkaf977_Supplemental_Files

## Data Availability

ChIP-seq and RNA-seq data generated in this study have been uploaded to GEO under accession number GSE199657: https://www.ncbi.nlm.nih.gov/geo/query/acc.cgi?acc=GSE199657. ChIP-seq data can be visualized on the UCSC genome browser: https://genome-asia.ucsc.edu/s/mshah/KLF3_WDR5%20R254A%20ChIP%2Dseq. Mass spectrometry data generated in this study have been uploaded on ProteomeXchange via the PRIDE database with the dataset identifier PXD033888: https://www.ebi.ac.uk/pride/archive/projects/PXD033888.
