# Supplementary material for: WDR5 serves in co-activation and influences genome targeting of KLF3
Source: Nucleic Acids Res. 2025 Sep 30;53(18):gkaf977. doi: 10.1093/nar/gkaf977 (PMC12481014; doi:10.1093/nar/gkaf977)
Supplement: gkaf977_Supplemental_Files [file gkaf977_supplemental_files.zip › 20250828_WDR5_supplementary_UPDATED.pdf]

Supplementary Figures

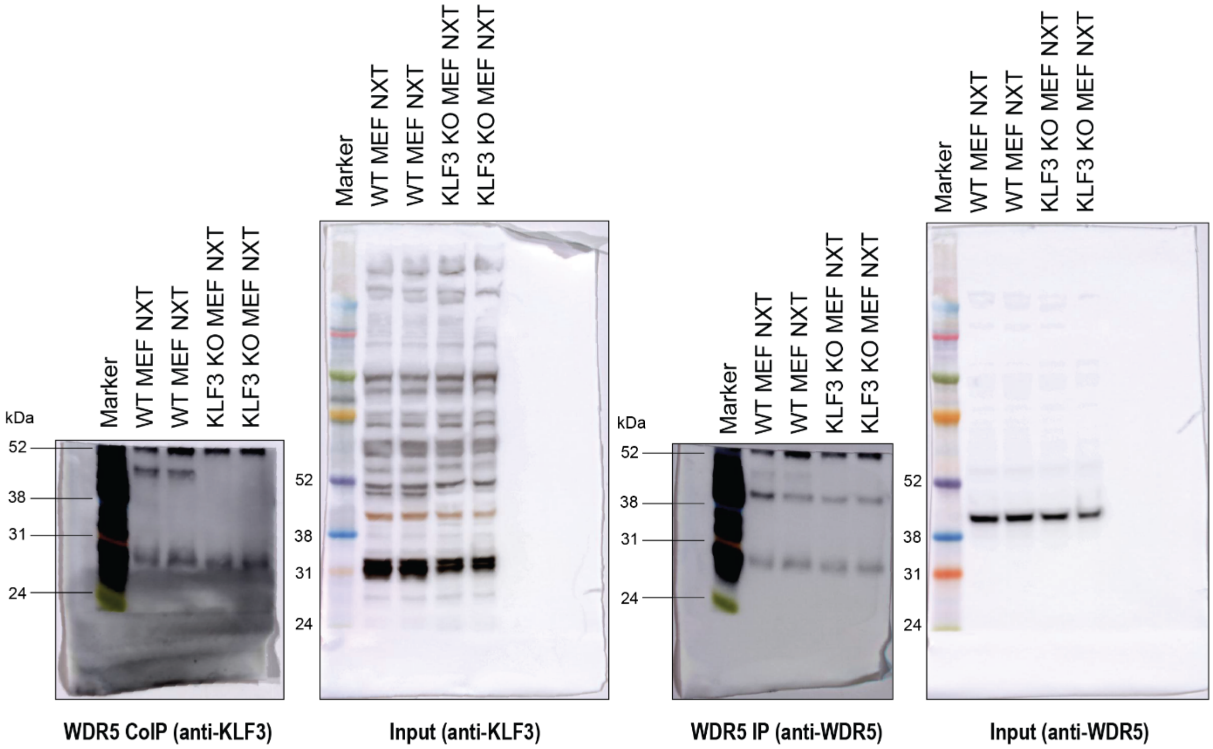

Figure S1 KLF3 and WDR5 association in MEF cells. Co-immunoprecipitation experiments were performed in WT or KLF3 KO MEF cells with endogenous WT KLF3 using anti-KLF3 or anti-WDR5 antibodies.

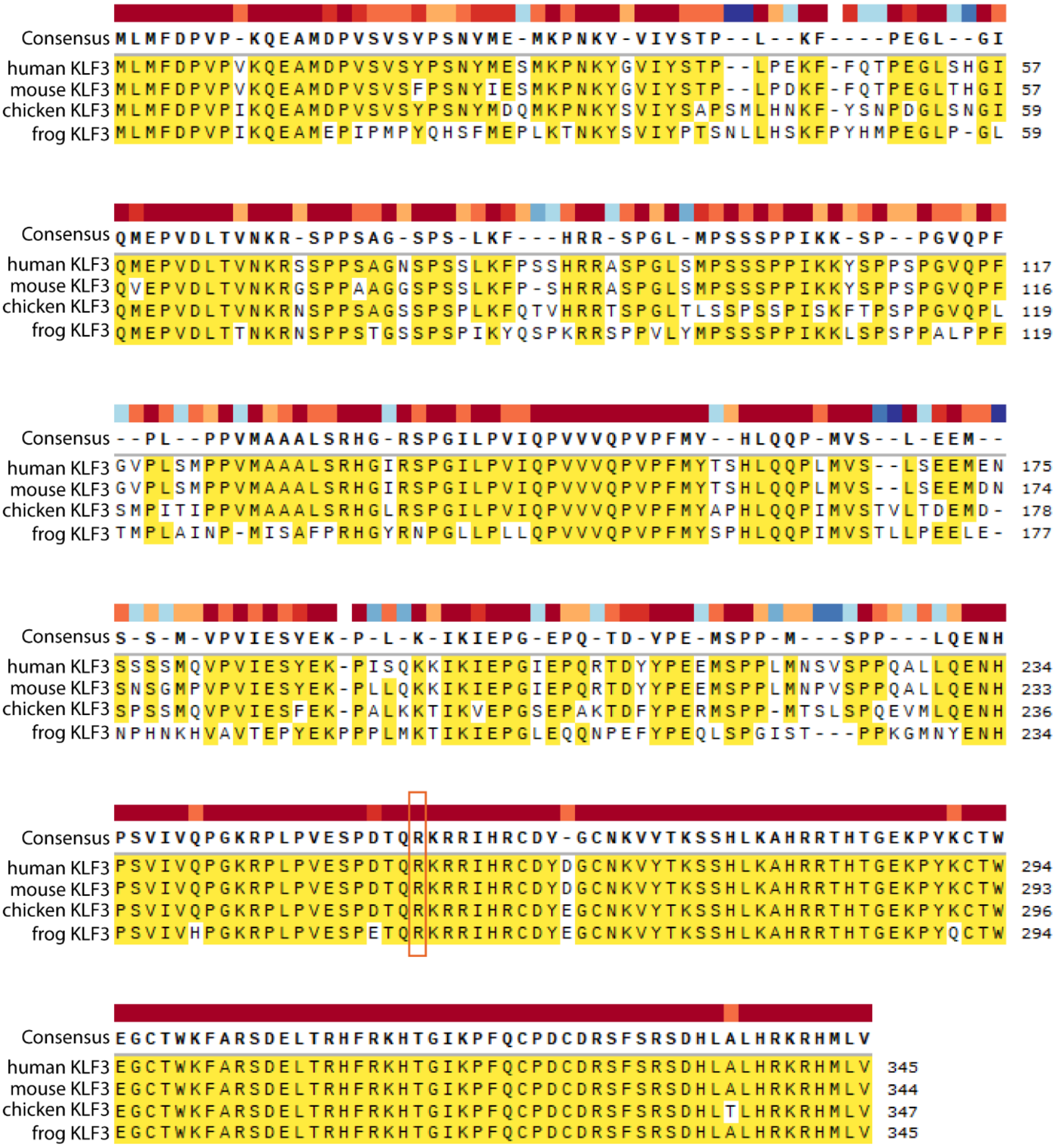

Figure S2 The conservation of the KLF3-WDR5 association interface among species. Alignment of KLF3 in four organisms: human, mouse, chicken and frog. The key WDR5 association residue (R254) is highlighted in the red box.

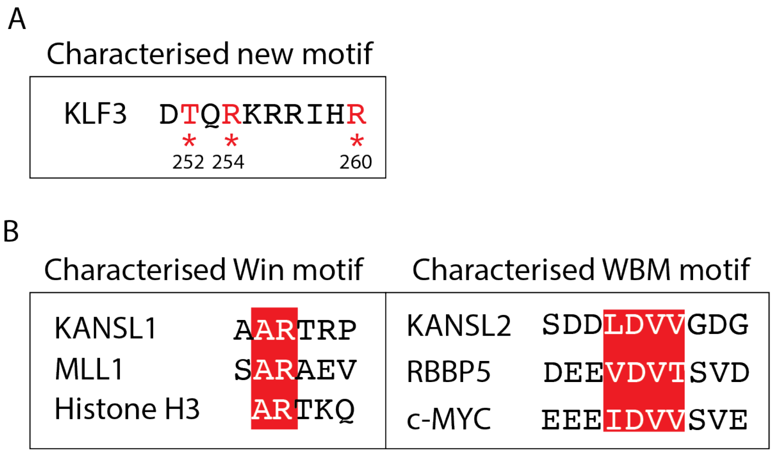

Figure S3 The WDR5 association motif in KLF3 is not conserved with WDR5 binding motifs in other WDR5 partner proteins A. The WDR5 association motif in KLF3 251-260; key amino acids are labelled with amino acid numbering and a red asterisk. B. The WDR5 binding motif (Win) found in KANSL1, MLL1 and Histone H3 (Ee et al. 2017, Song & Kingston 2008, Trievel & Shilatifard 2009) and WDR5 binding motif (WBM) found in KANSL2, RBBP5 and c-MYC (Odho et al. 2010, Thomas et al. 2015).

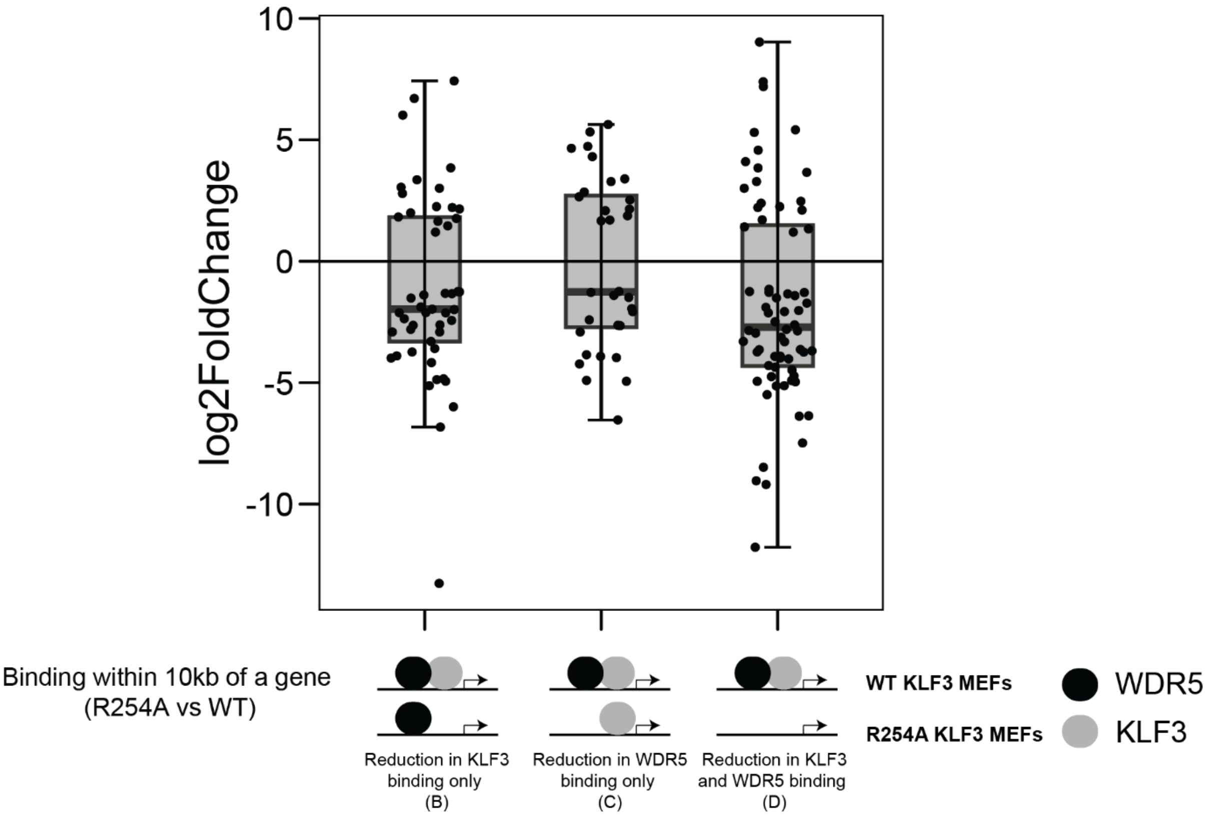

Figure S4 Loss of distal KLF3 and/or WDR5 binding led to a downregulation of gene expression. The average fold change (log2) of differentially expressed genes (p.adj < 0.05, R254A KLF3 MEFs vs WT KLF3 MEFs) that had both KLF3 and WDR5 bound within 10kb of the TSS and where there was a reduction in binding of either protein.

Supplementary Tables

Supplementary Table 1 Full List of candidate proteins found in CoIP/MS from HEK293 cells stably expressing KLF3-FD-V5. Peptides found in the negative control empty vector cells were disregarded. The number indicates the number of unique peptides identified that map to that protein. N/A indicates that the protein was not found.

Supplementary References

Ee L-S, McCannell KN, Tang Y, Fernandes N, Hardy WR, et al. 2017. An Embryonic Stem Cell-Specific NuRD Complex Functions through Interaction with WDR5. *Stem Cell Rep.* 8(6):1488–1496

Odho Z, Southall SM, Wilson JR. 2010. Characterization of a novel WDR5-binding site that recruits RbBP5 through a conserved motif to enhance methylation of histone H3 lysine 4 by mixed lineage leukemia protein-1. *J. Biol. Chem.* 285(43):32967–32976

Song J-J, Kingston RE. 2008. WDR5 interacts with mixed lineage leukemia (MLL) protein via the histone H3-binding pocket. *J. Biol. Chem.* 283(50):35258–35264

Thomas LR, Wang Q, Grieb BC, Phan J, Foshage AM, et al. 2015. Interaction with WDR5 promotes target gene recognition and tumorigenesis by MYC. *Mol. Cell.* 58(3):440–452

Trievel RC, Shilatifard A. 2009. WDR5, a complexed protein. *Nat. Struct. Mol. Biol.* 16(7):678–680
